# Pulmonary hemodynamics and effects of phosphodiesterase type 5 inhibition in heart failure: a meta-analysis of randomized trials

**DOI:** 10.1186/s12872-017-0576-4

**Published:** 2017-06-12

**Authors:** In-Chang Hwang, Yong-Jin Kim, Jun-Bean Park, Yeonyee E. Yoon, Seung-Pyo Lee, Hyung-Kwan Kim, Goo-Yeong Cho, Dae-Won Sohn

**Affiliations:** 1 Cardiovascular Center and Department of Internal Medicine, Seoul National University Hospital and Seoul National University College of Medicine, 101 Daehak-ro, Jongno-gu, Seoul, Republic of Korea; 2 Division of Cardiology, Department of Internal Medicine, Seoul National University Bundang Hospital, Seongnam-si, Gyeonggi-do Republic of Korea

**Keywords:** Phosphodiesterase 5 inhibitor, Heart failure, Pulmonary hypertension, Randomized controlled trial, Meta-analysis

## Abstract

**Background:**

Previous studies suggested that phosphodiesterase 5 inhibitors (PDE5i) have a beneficial effect in patients with heart failure (HF), although the results were inconsistent. We performed a meta-analysis to evaluate the effect of PDE5i in HF patients, and investigated the relationship between PDE5i effects and pulmonary hemodynamics.

**Method:**

We searched PubMed, EMBASE and the Cochrane Library for randomized controlled trials (RCTs) that compared PDE5i with placebo in HF with reduced ejection fraction (HFrEF) or HF with preserved EF (HFpEF). PDE5i effects were interpolated according to baseline pulmonary arterial pressure (PAP) or according to changes in PAP after PDE5i treatment.

**Results:**

Thirteen RCTs enrolling 898 HF patients, and two sub-analysis studies with different study outcomes, were included in the meta-analysis. Among patients with HFrEF, PDE5i improved peak VO_2_ (mean difference [MD], 3.76 mL/min/kg; 95% confidence interval [CI], 3.27 to 4.25; *P* < 0.00001), VE/VCO_2_ slope (MD, −6.04; 95% CI, −7.45 to −4.64; *P* < 0.00001), LVEF (MD, 4.30%; 95% CI, 2.18 to 6.42; *P* < 0.0001), and pulmonary vascular resistance (MD, −80.74 dyn·sec/cm^5^; 95% CI, −110.69 to −50.79; *P* < 0.00001). The effects of PDE5i in patients with HFpEF were heterogeneous. Meta-regression analyses indicated that the beneficial effect of PDE5i was related to the baseline PAP as well as the extent of PDE5i-mediated PAP decrease.

**Conclusion:**

PDE5i improved pulmonary hemodynamics and exercise capacity in patients with HFrEF, but not in HFpEF. The relationship between the benefits by PDE5i with the baseline PAP and the changes in PAP indicates the therapeutic potential of PDE5i in HF according to pulmonary hemodynamics.

**Electronic supplementary material:**

The online version of this article (doi:10.1186/s12872-017-0576-4) contains supplementary material, which is available to authorized users.

## Background

Despite considerable progresses in management of heart failure (HF), many patients demonstrate intractable symptoms even after the application of available therapeutic options [1]. Symptoms of dyspnea in HF are largely attributable to congestion in the pulmonary vasculature and pulmonary hypertension (PH) [2]. Epidemiologic studies indicate that 50–70% of patients with HF also have PH, defined as PH in left heart disease (PH-LHD) [3].

A number of clinical trials confirmed that the treatment with phosphodiesterase type 5 inhibitors (PDE5i) improved pulmonary hemodynamics and associated symptoms in patients with HF with reduced ejection fraction (HFrEF) [3–11]. However, the benefits of PDE5i in HF with preserved ejection fraction (HFpEF) were inconsistent [12–15]. It has been suggested that the recent disappointing results of PDE5i for patients with HFpEF are attributable to the selective effect of PDE5i on the pre-capillary pulmonary component [5, 16]. In other words, the benefit of PDE5i in treating HF may originate from its hemodynamic effect for the combined post- and pre-capillary PH (Cpc-PH), but not for the isolated post-capillary PH (Ipc-PH).

Given the growing evidence in support of the concept of PH-LHD and the suggested pre-capillary pulmonary selectivity of PDE5i, the relationship between the effect of PDE5i and pulmonary hemodynamics is of clinical importance. Therefore, we performed a meta-analysis of randomized clinical trials (RCTs) to evaluate the effects of PDE5i among patients with HF. We also investigated the relationship between PDE5i effects and pulmonary hemodynamics in this population.

## Methods

We performed a meta-analysis of the available published RCTs investigating the effects of PDE5i on HF. This study was conducted in accordance with the PRISMA statement [17].

### Search strategy and selection criteria

We searched PubMed, EMBASE, and the Cochrane Library for all published randomized clinical trials on the effects of PDE5i in HF patients up to the third week of April 2016. We also manually screened the reference lists from identified trials and review reports for inclusion of all relevant studies. The following keywords were used to search for the published clinical trials: *“heart failure”, “sildenafil”, “vardenafil”, “tadalafil”, “avanafil”, “udenafil”, “phosphodiesterase 5 inhibitors”, “phosphodiesterase type 5 inhibitors”, “PDE5 inhibitors”, “cardiac dysfunction”,* and *“pulmonary hypertension”*.

RCTs that assigned a PDE5 inhibitor or placebo to patients with HF were included. The following exclusion criteria were used: (1) treatment duration <4 weeks, (2) no access to full text articles, and (3) duplicate publications. However, duplicate publications from an original RCT that had different study outcomes were included in a mutually exclusive manner, to increase sensitivity and to avoid potential bias or exaggeration of the efficacy of a specific intervention [18, 19]. Any disagreements between the authors regarding the eligibility of a study were resolved by discussion.

### Data extraction and quality assessment

Two investigators (I.-C.H and Y.-J.K) independently extracted data from and assessed the validity of each trial. The following data were extracted from each trial: study design, number of patients, baseline patient characteristics, nature of intervention, assessment of pulmonary hemodynamics and exercise capacity, and clinical outcomes. The included RCTs were assessed for quality (Additional file 1: Figure S1).

### Outcomes and relation analysis

The included RCTs were assessed for the following outcomes: exercise capacity (peak VO_2_, VE/VCO_2_ slope, 6-minutes walking distance [6MWD]), cardiac performance (left ventricular ejection fraction [LVEF], cardiac index, and cardiac output), diastolic function (E/e’ ratio), and pulmonary resistance (mean pulmonary arterial pressure [mPAP], pulmonary arterial systolic pressure [PASP], pulmonary vascular resistance [PVR]). Clinical outcomes were assessed as all-cause death and hospitalization. Safety of PDE5i among HF patients was evaluated with the following outcomes: adverse event, heart rate (HR), systolic blood pressure (SBP), diastolic blood pressure (DBP), and mean arterial pressure (MAP).

PH was defined as an increase in mPAP ≥25 mmHg at rest. Post-capillary PH was defined as both mPAP ≥25 mmHg and pulmonary capillary wedge pressure (PCWP) >15 mmHg [20]. Post-capillary PH was further divided into the following two categories: Ipc-PH, which was defined as diastolic pulmonary gradient (DPG; diastolic PAP – PCWP) <7 mmHg and/or transpulmonary gradient (TPG; mPAP – PCWP) <12 mmHg; and Cpc-PH, defined as an increase in DPG ≥7 mmHg and/or TPG ≥12 mmHg.

### Statistical analysis

We assessed the effect of PDE5i in the following phenotypic subgroups; HFrEF and HFpEF. We used Review Manager 5.0 (Cochrane Collaboration, Oxford, UK) to analyze the collected data and to compare the data of the treatment group with that of the placebo group. If several studies were published from a single RCT, we only included the duplicate studies reporting different outcomes, and analyzed the data from each study in a mutually exclusive manner. If the study outcomes were presented in both of the duplicated studies from a single RCT, we used the values from the initial publication.

The effect size was calculated by the difference between the means of the treatment group and control group at the end of the intervention. If the values at the end of the intervention were not provided, we used the changes in values from baseline [18]. For studies that reported data as median values with interquartile ranges (IQR), we used the median values as the means, and converted the IQRs into standard deviations by dividing by 1.35, as recommended [18]. Outcomes were analyzed as continuous and dichotomized variables using a fixed model or a random effect model, and the results were reported as mean difference (MD) or risk ratio (RR) with 95% confidence interval (CI), respectively. The statistical strength was evaluated by the overall effect size (*Z*) and heterogeneity index (*I* ^*2*^). For the pooled results with significant heterogeneity, we performed sensitivity analyses by omitting one study at a time to establish the contribution of each study to the effect size.

Meta-regression analyses were performed to compare the therapeutic effects of PDE5i with pulmonary hemodynamic status. Mean or median values of pulmonary hemodynamics parameters of the included RCTs were compared with the changes in exercise capacity and cardiac performance, using age and proportion of male sex of each trial as covariates. In these meta-regression analyses, the PASP values determined by echocardiography were converted to mPAP using the following equation: mPAP = 0.61 × PASP +2.0 mmHg [21]. Considering that the RELAX trial had a larger study population than other RCTs, we performed a sensitivity analysis by omitting the RELAX trial, to assess whether the RELAX trial had significant influence on the findings of meta-regression analysis. Meta-regression analyses and influence analysis were performed using Stata/IC 11.0 (Stata Corporation, College Station, Texas, USA) and R version 3.2.5 (http://www.r-project.org).

## Results

### Searching results and study selection

**Fig. 1:**
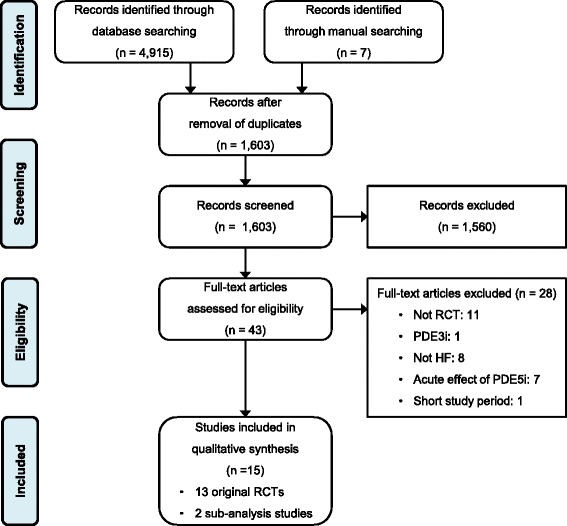
Flow diagram of study selection Abbreviations: RCT, randomized clinical trial; PDE3i, phosphodiesterase type 3 inhibitor; HF, heart failure

We identified 13 RCTs that were compatible with our selection criteria, and two sub-analysis studies that reported different outcomes than those presented in the original RCTs; *Lewis GD* et al. published a sub-analysis of a previous study [5, 22], and *Borlaug BA* et al. published a sub-analysis of the RELAX trial (Fig. 1) [13, 14]. Nine RCTs and one sub-analysis study enrolled 569 patients with HFrEF, and four RCTs and one sub-analysis study enrolled 329 patients with HFpEF (Table 1) [12–14, 23]. A total of 898 patients with HF were enrolled in the selected studies: 429 patients were assigned to sildenafil (with 428 patients assigned to placebo), and 21 patients were assigned to udenafil (with 20 patients assigned to placebo).

**Table 1:** Baseline Characteristics of Included RCTs

Study	Individuals randomized (n; PDE5i / Placebo)	Intervention		Inclusion criteria	Entry criteria		Male (%; PDE5i / Placebo)	Age (yrs; PDE5i / Placebo)	LVEF (%; PDE5i / Placebo)	mPAP (mmHg; PDE5i / Placebo)	PASP (mmHg; PDE5i / Placebo)	Follow-up duration (months)	Outcome measures
		Drug	Dosage		NYHA	LVEF							
Webster LJ et al., 2004 [26]	35/35	Sildenafil	50 mg qd	CHF (HFrEF)	II–III	-	100/100	60 (total)^a^	26 (total)^a^	-	-	1.5	International Index of Erectile Function
Katz SD et al., 2005 [27]	60/72	Sildenafil	25 / 50 / 100 mg	CHF (HFrEF) with ED	I–III	≤40%	100/100	60/60	33/30	-	-	3	International Index of Erectile Function
Guazzi M et al., 2007 [6]	23/23	Sildenafil	50 mg bid	HFrEF	II–III	≤45%	100/100	62/63	30.6/31.9	-	34/32	6	CPET, EchoCG, FMD
Lewis GD et al., 2007 [5]	17/17	Sildenafil	25 to 75 mg tid	HFrEF with PH	II–IV	<40%	82/88	54/62	19/20	30/33	-	3	CPET: peak VO_**2**_
Behling A et al., 2008	11/8	Sildenafil	50 mg tid	HFrEF	I–III	≤40%	82/50	45/53	27/39	-	56/62	1	CPET, EchoCG, FBF
Lewis GD et al., 2008 [22]	15/15	Sildenafil	25 to 75 mg tid	HFrEF with PH^b^	II–IV	<40%	90	58	26/28	30/33	-	3	CPET, Cardiac Cath, ventriculography
Guazzi M et al., 2011 [11]	23/22	Sildenafil	50 mg tid	HFrEF	II–III	<40%	100/100	60/61	29.5/30.2	-	37/38	12	EchoCG, CPET, BNP, QoL
Guazzi M et al., 2012 [7]	16/16	Sildenafil	50 mg tid	HFrEF with PH and EOB	III–IV	<45%	100/100	66/68	29/28	35/34	-	12	CPET, Cardiac Cath
Amin A et al., 2013 [8]	53/53	Sildenafil	25 mg bid for first 2 weeks; 50 mg tid for next 10 weeks	HFrEF	II–III	<35%	72/75	51/51	-	-	-	3	BP, NYHA, 6MWD
Kim KH et al., 2015 [9]	21/20	Udenafil	50 mg bid for first 4 weeks; 100 mg bid for next 8 weeks	HFrEF	II–IV	≤40%	76/60	62/65	30/29	-	41/43	3	EchoCG, CPET
Guazzi M et al., 2011 [12]	22/22	Sildenafil	50 mg tid	HFpEF with PH	II–IV	≥50%	77/82	72/73	60/60	39/37	55/52	12	EchoCG, Cardiac Cath, QoL
Redfield MM et al., 2013 [13]	113/103	Sildenafil	20 mg tid for first 12 weeks; 60 mg tid for next 12 weeks	HFpEF (RELAX trial)	II–IV	≥50%	57/47	68/69	60/60	-	41/41	6	EchoCG, CMRI, CPET, 6MWD
Borlaug BA et al., 2015 [14]	23/25	Sildenafil	20 mg tid for first 12 weeks; 60 mg tid for next 12 weeks	HFpEF (RELAX trial)^c^	II–IV	≥50%	39/44	69/71	60/60	-	28/30	6	EchoCG, CMRI, CPET, 6MWD, Radial applanation tonometry
Hoendermis ES et al., 2015 [15]	21/22	Sildenafil	20 mg tid for first 2 weeks; 60 mg tid for next 10 weeks	HFpEF with PH	II–III	≥45%	23/35	72/76	58/58	35/35	52/51	3	Cardiac Cath, CPET, EchoCG
Andersen MJ et al., 2013 [23]	35/35	Sildenafil	40 mg tid	Diastolic dysfunction after MI	-	≥45%	89/86	63/62	55/56	19/20	26/27	2	EchoCG, Cardiac cath, CPET, 6MWT

*Abbreviations*: *CHF* congestive heart failure, *HFrEF* heart failure with reduced ejection fraction, *HFpEF* heart failure with preserved ejection fraction, *PH* pulmonary hypertension, *EOB* exercise oscillatory breathing, *MI* myocardial infarction, *NYHA* New York Heart Association, *LVEF* left ventricular ejection fraction, *PDE5i* phosphodiesterase type 5 inhibitor, *mPAP* mean pulmonary arterial pressure, *PASP* pulmonary artery systolic pressure, *CPET* cardiopulmonary exercise test, *EchoCG* echocardiography, *FMD* flow-mediated dilatation, *BNP* B-natriuretic peptide, *QoL* quality of life, *BP* blood pressure, *6MWD* 6-min walking distance, *CMR* cardiac magnetic resonance

^a^ Mean of total study population

^b^ *Lewis GD* et al. 2008 [22] was a sub-analysis of '*Lewis GD* et al. 2007 [5]'

^c^ *Borlaug BA* et al. 2015 [14] was a sub-analysis of '*Redfield* et al. 2013 [13]', the RELAX trial

The Sildenafil and Diastolic Dysfunction After Acute Myocardial Infarction (SIDAMI) trial by *Andersen* et al. included patients with diastolic dysfunction and preserved EF after myocardial infarction [23]. Although this study did not enroll patients with definite symptomatic HFpEF, it was included in our meta-analysis because the enrolled patients represent the spectrum of HFpEF, and the hemodynamic abnormalities caused by diastolic dysfunction would drive the progression of symptomatic HFpEF [24, 25]. Moreover, the baseline mPAP values at peak exercise of the SIDAMI trial were 49 ± 10 mmHg in the placebo group and 44 ± 9 mmHg in the sildenafil, indicating that most of the study participants had exercise-induced PH [2]. Given the presence of both exercise-induced PH and diastolic dysfunction, we decided to include the SIDAMI trial by *Andersen* et al. in our meta-analysis.

### Hard endpoint and adverse event

**Fig. 2:**
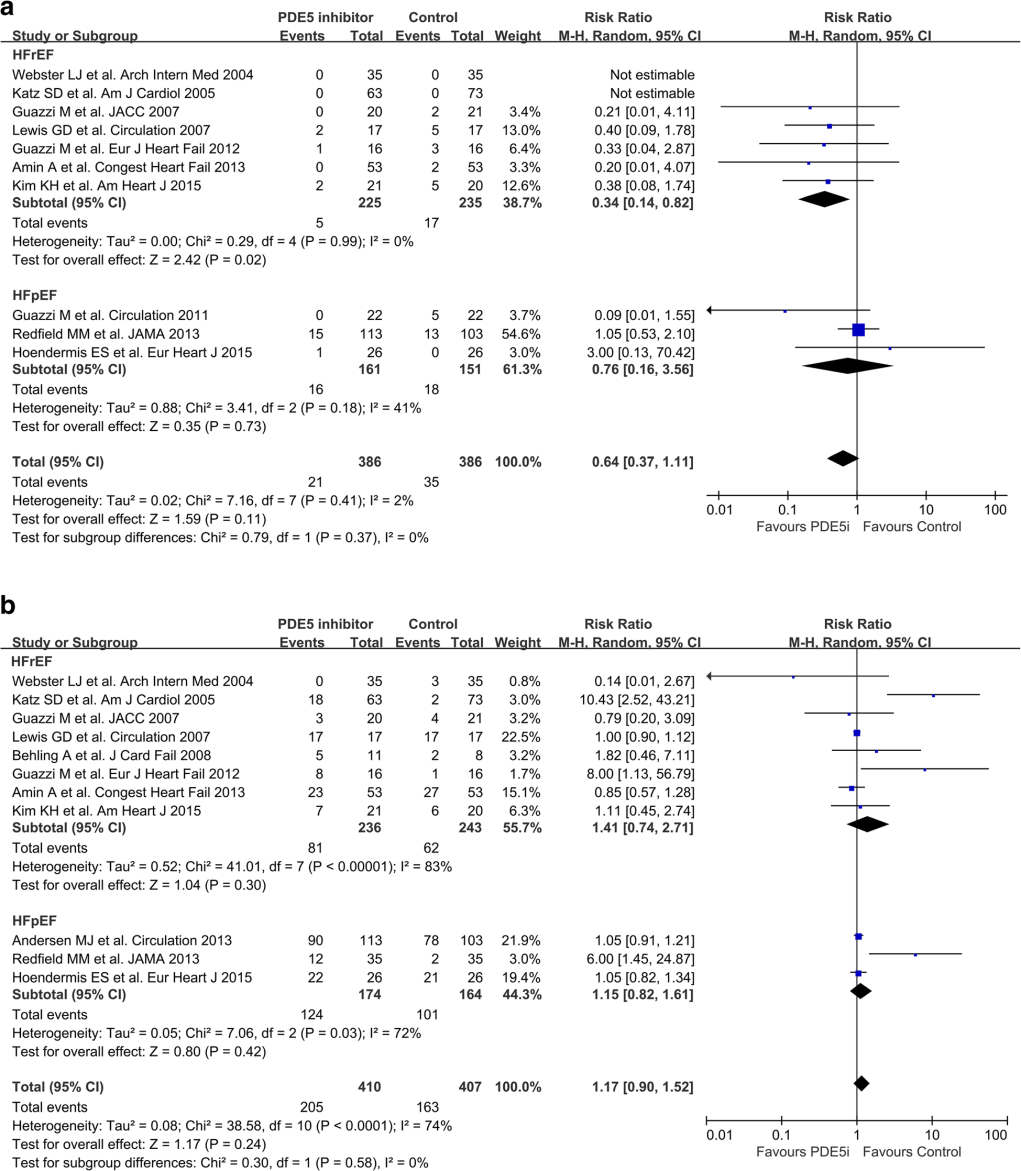
Clinical outcomes and adverse events. Forest plot of the pooled weighted risk ratio for the occurrence of (**a**) death or hospitalization and (**b**) adverse events. Abbreviations: HFrEF, heart failure with reduced ejection fraction; HFpEF, heart failure with preserved ejection fraction; CI, confidence interval

Seven RCTs of HFrEF reported clinical outcomes, with five hospitalization events occurring in the PDE5i arm and 17 occurring in the control arm [5–9, 26, 27]. These results indicate a significant benefit conferred by PDE5i against hospitalization (RR, 0.340; 95% CI, 0.140 to 0.820; *P* = 0.02; Fig. 2a). Two RCTs of HFpEF reported 15 hospitalization events occurring in the PDE5i arm and 18 occurring in the control arm (RR, 0.450; 95% CI, 0.040 to 4.86; *P* = 0.51). During the follow-up period, five deaths were reported [8, 13]. The occurrence of adverse events in these two studies did not significantly differ between the PDE5i arm and the control arm (Fig. 2b). The use of PDE5i in patients with HF did not significantly affect SBP, DBP, MAP and HR (Additional file 2: Figure S2).

### Exercise capacity and cardiac performance

**Fig. 3:**
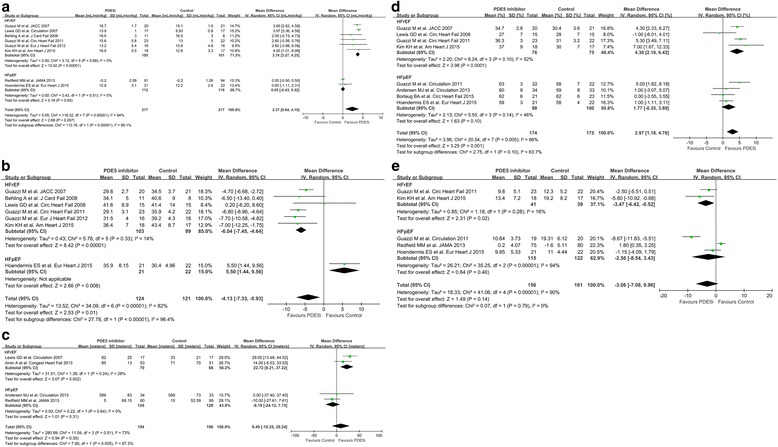
Effect of PDE5i on exercise capacity and cardiac performance. Forest plot of the pooled weighted mean differences of (**a**) peak VO_2_ (mL/min/kg), (**b**) ventilatory efficiency (VE/VCO_2_ slope), (**c**) 6MWD (meters), (**d**) LVEF (%), and (**e**) Mitral annular E/e’ ratio. Abbreviations: 6MWD, 6-min walking distance; LVEF, left ventricular ejection fraction; other abbreviations as in Fig. 2

The use of PDE5i significantly improved exercise capacity in patients with HFrEF (Fig. 3). In HFrEF patients, the use of PDE5i improved peak VO_2_ (MD, 3.76; 95% CI; 3.27 to 4.25; *P* < 0.00001; Fig. 3a), ventilatory efficiency (VE/VCO_2_ slope; MD, −6.04; 95% CI, −7.45 to −4.64; *P* < 0.00001; Fig. 3b), and 6MWD (MD, 22.72; 95% CI, 8.21 to 37.22; *P* = 0.002; Fig. 3c). By contrast, RCTs of patients with HFpEF did not demonstrate any benefit of PDE5i use on exercise capacity as measured by cardiopulmonary exercise test or 6MWD.

For patients with HFrEF, the use of PDE5i significantly improved LVEF (MD, 4.30%; 95% CI, 2.18 to 6.42; *P* < 0.0001; Fig. 3d). The use of PDE5i for HFpEF resulted in a tendency for improved LVEF (MD, 1.77%; 95% CI, −0.35 to 3.89; *P* = 0.10). PDE5i improved cardiac output in HFrEF patients, and tended to increase cardiac index in HFpEF patients (Additional file 2: Figure S3). The use of PDE5i in HFrEF decreased mitral annular E/e’ ratio, but did not significantly affect those values in HFpEF (Fig. 3e).

### Pulmonary resistance and pulmonary pressures

**Fig. 4:**
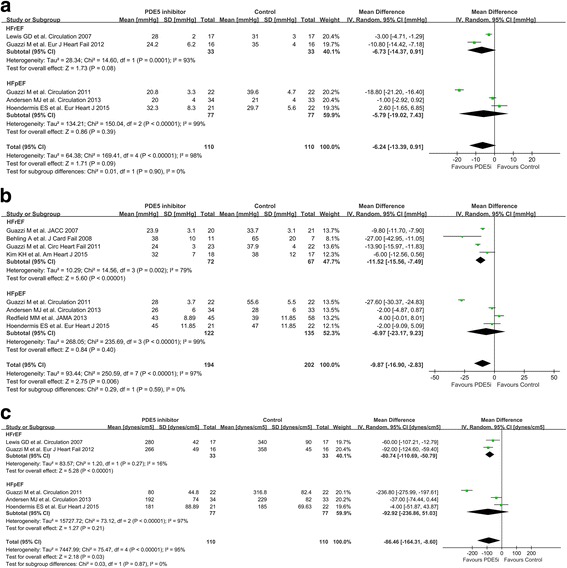
Effect of PDE5i on pulmonary hemodynamics. Forest plot of the pooled weighted mean differences of (**a**) mPAP (mmHg), (**b**) PASP (mmHg), and (**c**) PVR (dyn·sec/cm^5^). Abbreviations: mPAP, mean pulmonary arterial pressure; PASP, pulmonary arterial systolic pressure; PVR, pulmonary vascular resistance; other abbreviations as in Fig. 2

For patients with HFrEF, PDE5i reduced mPAP (MD, −6.73 mmHg; 95% CI, −14.37 to 0.91; *P* = 0.08; Fig. 4a), PASP (MD, −11.52 mmHg; 95% CI, −15.56 to −7.49; *P* < 0.00001; Fig. 4b), and PVR (MD, −80.74 dyn·sec/cm^5^; 95% CI, −110.69 to −50.79; *P* < 0.00001; Fig. 4c). The PDE5i-mediated improvement in pulmonary hemodynamic parameters for patients with HFrEF was concordant among the RCTs. A weak association between the use of PDE5i and improvement in pulmonary hemodynamics was observed for patients with HFpEF; however, the included RCTs showed heterogeneous results.

Among the RCTs of HFpEF, the study by *Guazzi M* et al. [11], reported significant benefits of PDE5i [12], however, the other studies of HFpEF reported contradicting results: there was no significant benefit by PDE5i in patients with HFpEF according to the RELAX trial [13, 14], and the other trials by *Andersen* et al. [23], and *Hoendermis* et al. [15]. In order to assess the influence from the RCT by *Guazzi M* et al. [11], we performed sensitivity analyses by omitting one study at a time (Additional file 3: Figure S4). The influence analysis showed that the RCT by *Guazzi M* et al. [12] had a significant influence on the pooled effect of PDE5i in HFpEF patients, whereas the omission of the other RCTs did not.

### Correlation between pulmonary hemodynamics and PDE5i effects

Five RCTs with one sub-analysis reported the findings from cardiac catheterization (Table 2). We utilized the measured or calculated values of mPAP, DPG, TPG, and PVR to identify three RCTs and one sub-analysis that primarily enrolled patients with Cpc-PH: Lewis et al. [5]; *Lewis GD* et al. [22]; *Guazzi M* et al. [11]; and *Guazzi M* et al. [7]. The use of PDE5i in these trials demonstrated the overall beneficial effects on exercise capacity, LV function, and pulmonary hemodynamics. The trials for HFrEF with probable Cpc-PH, as indicated by elevated TPG values, showed consistent improvement in exercise capacity and reduction in pulmonary pressures [5, 7, 22]. Also, one RCT of patients with HFpEF and with Cpc-PH, by *Guazzi M* et al. [11], reported a significant benefit of PDE5i treatment for LV function and pulmonary hemodynamics [12]. By contrast, RCTs of patients with HFpEF and low levels of PAP at baseline reported no observed benefit of PDE5i treatment for patients with HFpEF without PH [13–15, 23].

**Table 2:**
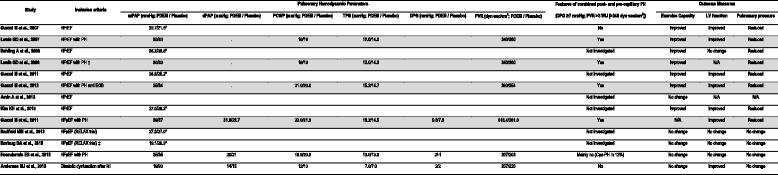
Differential Impact of PDE5 inhibitors according to Pulmonary Hemodynamics

Improvement in exercise capacity was assessed using the study outcomes on peak VO_2_ and VE/VCO_2_ slope by CPET, or 6MWD. Improvement in LV function was assessed using the study outcomes on LVEF, cardiac output, and cardiac index. Reduction in pulmonary pressures was assessed using the study outcomes on mPAP, PCWP and PVR by cardiac catheterization, or PASP by echocardiogram. The grey-colored rows indicate the trials for HF with probable Cpc-PH, as suggested by elevated TPG and DPG values, showing consistent improvements in exercise capacity, LV function, and pulmonary hemodynamics

*Abbreviations*: *HFrEF* heart failure with reduced ejection fraction, *HFpEF* heart failure with preserved ejection fraction, *PH* pulmonary hypertension, *EOB* exercise oscillatory breathing, *MI* myocardial infarction, *mPAP* mean pulmonary arterial pressure, *dPAP* diastolic pulmonary arterial pressure, *PCWP* pulmonary capillary wedge pressure, *TPG* transpulmonary gradient, *DPG* diastolic pulmonary gradient, *LV* left ventricle, *N/A* not applicable

^a^ Converted from echocardiographic PASP by the following equation: mPAP (mmHg) = (0.61 × PASP [mmHg]) + 2 mmHg [21]

^b^ Sub-analysis of ‘*Lewis GD* et al. [5]’ [22]

^c^ Sub-analysis of ‘*Redfield* et al. [13]’, the RELAX trial [14]

**Fig. 5:**
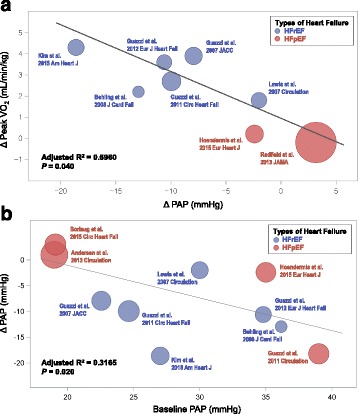
Associations between pulmonary hemodynamics and PDE5i effects. **a** Interpolated relationships from meta-regression analysis of the PDE5i-mediated reduction in PAP levels and the changes in peak VO_2_ (Δpeak VO_2_, mL/min/kg). **b** Interpolated relationships between the baseline PAP levels and the PDE5i-mediated reduction in PAP levels. *Blue circles* indicate the results from HFrEF trials; *red circles* indicate the results from HFpEF trials. Ages and proportions of male sex were used as covariates. Abbreviations: PAP, pulmonary arterial pressure; other abbreviations as in Fig. 2

The relationship between changes in mPAP and changes in peak VO_2_ is presented in Fig. 5a. The PDE5i-mediated decrease in mPAP was significantly correlated with increased peak VO_2_ levels, after adjusting for age and sex (Δpeak VO_2_; adjusted R^2^ = 0.6960; *P* = 0.040). Considering the significantly larger study population of the RELAX trial by *Redfield MM* et al. [13], we performed a sensitivity analysis by omitting the RELAX trial from the meta-regression analysis. The overall relationship between the changes in mPAP and the changes in peak VO_2_ remained significant when the RELAX trial was omitted from the analysis (adjusted R^2^ = 0.5345; *P* = 0.048) [13].

We also interpolated baseline mPAP with PDE5i-mediated changes in mPAP, and showed that RCTs with higher baseline mPAP had significantly larger reductions in mPAP values even after adjusting for covariates (adjusted R^2^ = 0.3165; *P* = 0.020; Fig. 5b). Given that the RCT by *Guazzi M* et al. [12] was the only study that reported benefits of PDE5i in HFpEF, a sensitivity analysis was performed. Of note, the omission of the RCT by *Guazzi M* et al. [12] did not significantly change the relationship between the baseline mPAP and the changes in mPAP by PDE5i (adjusted R^2^ = 0.3069; *P* = 0.019).

## Discussion

This meta-analysis shows that the use of PDE5i in patients with HFrEF improves exercise capacity, cardiac performance, and pulmonary hemodynamics. The benefit of PDE5i on exercise capacity was related to the extent of PDE5i-mediated decrease in PAP. Although the RCTs of HFpEF reported heterogeneous results, the effects of PDE5i in patients with HFpEF were consistent with the interpolated relationship between PAP and outcome parameters. Our findings suggest the therapeutic potential of PDE5i in patients with HF, in relation with pulmonary hemodynamics.

### PDE5i treatment is beneficial in HFrEF patients

In this meta-analysis, we observed significant and consistent benefits conferred by PDE5i treatment for patients with HFrEF. These findings are in line with the results of previous meta-analysis by *Zhuang* et al. [4], but we further extended the previous study and provided more comprehensive results on the changes in exercise capacity and hemodynamic parameters by including the most recently published RCTs. Therefore, we successfully showed the therapeutic effect of PDE5i in HF patients in relation with the baseline pulmonary hemodynamics.

More importantly, we summarized the occurrence of clinical events including death and hospitalization from the previous RCTs. Our results also suggest that PDE5i would reduce the occurrence of death and hospitalization in patients with HFrEF, whereas it does not increase adverse events or affect BP and HR. The interpretation of this result needs caution, because the number of RCTs that reported clinical outcomes was small, and the follow-up duration of each trial was not longer than 12 months. However, this finding can be a meaningful result, when interpreted in relation to the benefits by PDE5i on the other study outcomes: the improved cardiac performance and pulmonary hemodynamics might result in the better prognosis.

Potential benefits of PDE5i in HF have been suggested from previous animal studies: PDE5i prevented myocardial dysfunction by anti-remodeling, anti-apoptotic and anti-inflammatory effects in various disease models of HF [28–30]. Our group developed a rat model of chronic myocardial regurgitation, and showed that the use of sildenafil significantly attenuated LV remodeling and prevented exercise intolerance, probably through the anti-apoptotic and anti-inflammatory effects [28]. Cardioprotective effect of sildenafil was supported in a chronic model of doxorubicin cardiotoxicity [31], as well as an ischemic cardiomyopathy model [32]. Another PDE5i, udenafil, also demonstrated cardioprotective effect in rats exposed to pressure-overload cardiac hypertrophy: udenafil prevented cardiac remodeling and improved exercise capacity and survival, through inhibition of fibrosis and apoptosis, and modulation of inflammatory cytokines in the hypertrophied myocardium [29]. These findings suggest that the long-term use of PDE5i in HF might be beneficial for myocardial reverse remodeling, improvement in LV function, and probably prevention of cardiovascular mortality. Until now, there has been no study to suggest the prognostic benefit of PDE5i in HFrEF patients, and our meta-analysis may serve as a hypothesis-generating study for future trials [1, 33].

Of note, among the 15 included RCTs, there was one RCT of udenafil for HFrEF that showed significant benefits on cardiac function and exercise capacity [9]. Together with the hemodynamic benefit and safety of udenafil reported in previous studies [29, 34–36], the use of udenafil could be a promising therapeutic measure in patients with HFrEF, when compared to sildenafil. Given the concerns on the differences in pharmacodynamic profile between the PDE5i medications and the potential adverse events, further trials are required for the use of other PDE5i, such as tadalafil, vardenafil, and avanafil, in HF patients.

### Inconclusive effect of PDE5i in HFpEF patients

The action mechanism of PDE5i in HF was suggested to be relaxation of pulmonary vessels and reverse remodeling effects on pulmonary vasculature and LV myocardium [37]. *Guazzi M* et al. reported the first RCT on the effect of PDE5i in HFpEF patients, and demonstrated significant improvement in LVEF and reduction in pulmonary resistance [12]. However, the following two RCTs of PDE5i in patients with HFpEF reported no significant benefits. The RELAX trial showed that the use of sildenafil in HFpEF patients was not associated with improved exercise capacity and cardiac performance [13]. Similarly, in a recent RCT by *Hoendermis* et al., sildenafil did not reduce pulmonary pressure or improve cardiac performance in patients with HFpEF [15].

Previous trials reported inconsistent results and the pooled effects are inconclusive in determining the effect of PDE5i in HFpEF. We also showed that the pooled effect of PDE5i needs to be interpreted with caution, considering the potential influence from the RCT by *Guazzi M* et al. [12] on the outcome measures. However, considering the pathophysiologic background of PH-LHD as well as the potential effect of PDE5i in this context, we attempted further analysis of the pooled results according to pulmonary hemodynamics.

### Pulmonary hemodynamics modulates the effect of PDE5i in HF patients

In HF patients, diastolic dysfunction leads to passive backward transmission of filling pressures, which results in PH. This condition is defined as Ipc-PH [3]. Among patients with HF and PH, some patients develop further increase in pulmonary pressures, defined as Cpc-PH, due to the combination of pulmonary vasoconstriction, decreased NO availability, increased endothelin expression, desensitization to natriuretic peptide-induced vasodilation, and vascular remodeling [2, 38]. The stage of Ipc-PH in HF patients indicates that the cause of PH is pure mechanical component, other than endothelial dysfunction or limited NO bioavailability [3]. By contrast, Cpc-PH is a more advanced stage in which NO availability and remodeling of pulmonary vasculature and LV have more important roles [39].

Our meta-regression analysis showed that the PDE5i-mediated PAP decrease had a linear interpolation relationship with the improvement in exercise capacity, and that the higher baseline PAP is an important determinant of the PDE5i-mediated benefits. Results of our sensitivity analysis also supported the significant association between the PDE5i-mediated improvements in PAP and exercise capacity. These findings are in accordance with the early study by *Lewis GD* et al., which showed direct correlations between baseline pulmonary resistance and PDE5i-mediated improvement in exercise capacity [5]. These results also suggest that the benefits of PDE5i in HF patients would be related to the degree of PH. Add to this, our data suggest that the use of PDE5i in HFpEF patients would be beneficial for those with higher baseline pulmonary pressure and whose pulmonary pressure could be reduced by PDE5i, namely, those with Cpc-PH. In particular, the beneficial effect of PDE5i in HFpEF reported by *Guazzi M* et al. may be attributable to the more advanced PH at baseline [12]. In the RELAX trial, however, the pulmonary pressure was not specified as an entry criterion, and participants had a mean of PASP 41 mmHg and calculated mPAP of 27 mmHg [13, 40]. Therefore, the lack of a beneficial effect of PDE5i in the RELAX trial may be associated with the relatively low pulmonary pressures and the small proportion of participants with Cpc-PH. Similarly, there were only six patients (12%) with Cpc-PH in the recent RCT by *Hoendermis* et al., which reported no beneficial effect of PDE5i [15].

Therefore, the heterogeneous results reported in the RCTs of HFpEF patients should not be regarded as the failure of PDE5i in this patient population, but rather that the data might be interpreted in consideration of the baseline PH stage of the study participants. In the present study, there was a significant relationship between the PDE5i-mediated PAP decrease and the baseline pulmonary pressure. This finding suggests that the suboptimal or nonsignificant effects of PDE5i in the RCTs of HFpEF could be related to the presence of Ipc-PH, but not attributable to the preserved LVEF. As an integration of previous trials, our meta-analysis suggests that the effect of PDE5i might be dependent to the pulmonary hemodynamics in HF patients, and that the patients with Cpc-PH could be benefited by PDE5i therapy. Moreover, our findings are in accordance with pathophysiologic background and recent concepts of PH-LHD: the Cpc-PH indicates a more advanced form of PH-LHD, and the increased PAP in Cpc-PH patients can be alleviated by PDE5i. Given the potential hemodynamic benefit conferred by PDE5i in Cpc-PH, we believe that further trials would provide more important relevance in the management of PH-LHD.

### Limitations

This study has several limitations in addition to the inherent methodological limitations of meta-analysis. First, the main study outcomes of our meta-analysis were not reported in all of the included RCTs, and cardiac catheterization was not performed in some RCTs. In our meta-analysis, the PASP values were converted to mPAP values, which might not be exactly proportional to PAP measurement by cardiac catheterization [21]. The lack of those outcome parameters limited the overall statistical power of this meta-analysis. Second, there is a possibility that the patients included in the prior study were duplicated in the following studies. Although we tried to minimize any potential influence from the duplicated patients, the interpretation and generalization of our findings need caution. Third, the study population of the RELAX trial was significantly larger than that of other trials; therefore, the results of HFpEF analyses might have been influenced by the RELAX trial results. Moreover, among the RCTs of HFpEF, there was only one study that reported positive findings favoring the use of PDE5i (*Guazzi M* et al. Circulation 2011) [12], whereas the other RCTs of HFpEF demonstrated negative results. Nevertheless, the inconsistent results of the HFpEF analysis can be explained by the interpolated relationship between pulmonary hemodynamics and PDE5i therapy, which showed overall concordance among the included RCTs of both HFrEF and HFpEF. Despite the potential influences from the larger study population of the RELAX trial and its limitation of not using pulmonary hemodynamics as an entry criterion, the integrated results support the therapeutic effect of PDE5i in HF patients with advanced PH. Also, our sensitivity analysis showed that the associations between the PDE5i-mediated potential benefits and pulmonary hemodynamics were not influenced from the RELAX trial or the RCT by *Guazzi M* et al., suggesting that our findings would have clinical implications. Fourth, the concept of PH-LHD with differentiation between Ipc-PH and Cpc-PH was not an inclusion criterion in the selected RCTs. Because the study participants of each RCT may have had different hemodynamic profiles, the simple interpolation analysis could not be applied to each individual patient. However, we showed the overall association between pulmonary hemodynamics and the effects of PDE5i, suggesting the potential application of PDE5i in PH-LHD. In addition, our findings imply that future trials on HFpEF need to consider the pulmonary hemodynamics not only as study endpoints but also as important eligibility criteria.

## Conclusions

The use of PDE5i in patients with HFrEF showed beneficial effects on pulmonary hemodynamics and exercise capacity. The use of PDE5i in patients with HFpEF showed conflicting results, however, the effects of PDE5i in HFpEF were consistent with the interpolated relationship between PAP values and improved outcome parameters. Our findings suggest a potential therapeutic role of PDE5i according to the pulmonary hemodynamics in HF patients.

## Additional files

Additional file 1: Figure S1. Quality assessment. **(A)** Risk of bias summary. **(B)** Risk of bias graph. (PPTX 76 kb)

Additional file 2: Figure S2. Effect of PDE5i on BP and HR. Forest plot of the pooled weighted mean differences of **(A)** SBP (mmHg), **(B)** DBP (mmHg), **(C)** MAP (mmHg), and **(D)** HR (beat per minute). Abbreviations: SBP, systolic blood pressure; DBP, diastolic blood pressure; MAP, mean arterial pressure; HR, heart rate. **Figure S3.** Effect of PDE5i on cardiac performance. Forest plot of the pooled weighted mean differences of **(A)** cardiac index (L/min/m^2^), and **(B)** cardiac output (L/min). (TIF 5850 kb)

Additional file 3: Figure S4. Influence analysis for the RCTs of HFpEF. Sensitivity analysis was performed to assess the potential influence of each RCT to the effect size of the RCTs of HFpEF. Pooled effects of PDE5i when each RCT was omitted were shown for **(A)** LVEF (%), **(B)** mPAP (mmHg), **(C)** PASP (mmHg), and **(D)** PVR (dyn·sec/cm^5^). The omission of the study by *Guazzi M* et al. [12] significantly changed the pooled effect size of PDE5i, suggesting that there was a substantial influence from the study by *Guazzi M* et al. on the overall outcome measures. Abbreviations: RCT, randomized controlled trial; HFpEF, heart failure with preserved ejection fraction; PDE5i, phosphodiesterase type 5 inhibitor; LVEF, left ventricular ejection fraction; mPAP, mean pulmonary artery pressure; PASP, pulmonary artery systolic pressure; PVR, pulmonary vascular resistance. (PPTX 588 kb)

## Abbreviations

6MWD: 6-minutes walking distance
Cpc-PH: Combined post- and pre-capillary pulmonary hypertension
DPG: Diastolic pulmonary gradient
HF: Heart failure
HFpEF: Heart failure with preserved ejection fraction
HFrEF: Heart failure with reduced ejection fraction
Ipc-PH: Isolated post-capillary pulmonary hypertension
LVEF: Left ventricular ejection fraction
MD: Mean difference
mPAP: mean pulmonary arterial pressure
PAP: Pulmonary arterial pressure
PASP: Pulmonary arterial systolic pressure
PCWP: Pulmonary capillary wedge pressure
PDE5i: Phosphodiesterase 5 inhibitors
PH: Pulmonary hypertension
PH-LHD: Pulmonary hypertension in left heart disease
PVR: Pulmonary vascular resistance
RCT: Randomized controlled trial
RR: Risk ratio
TPG: Transpulmonary gradient
