# Supplementary figures and images for: Pulmonary hemodynamics and effects of phosphodiesterase type 5 inhibition in heart failure: a meta-analysis of randomized trials
Source: BMC Cardiovasc Disord. 2017 Jun 12;17:150. doi: 10.1186/s12872-017-0576-4 (PMC5468951; doi:10.1186/s12872-017-0576-4)

## Slide 1
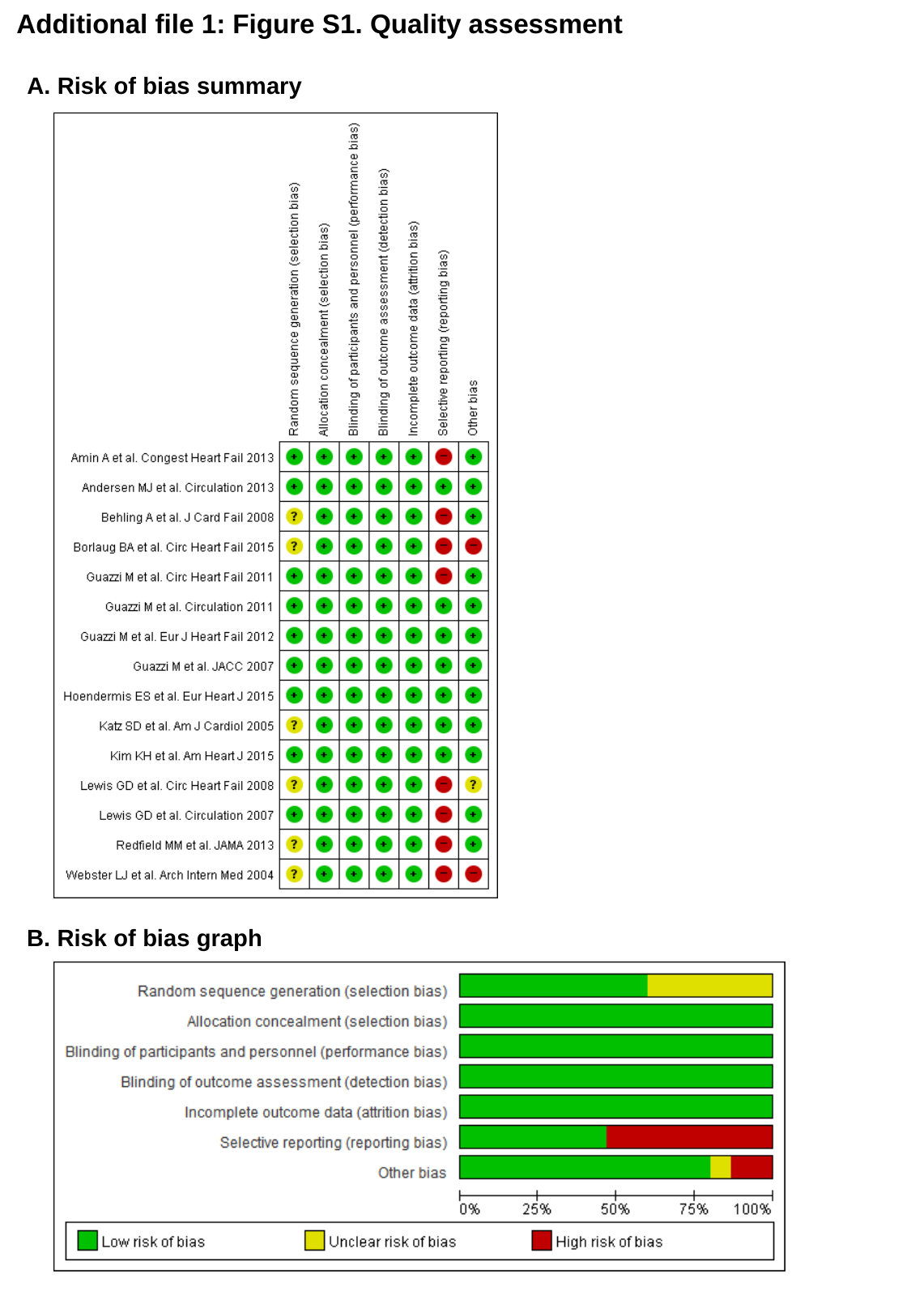

Additional file 1: Figure S1. Quality assessment
A. Risk of bias summary
B. Risk of bias graph

Supplement: Supplementary file 1 — Quality assessment. (A) Risk of bias summary. (B) Risk of bias graph. (PPTX 76 kb) [file 12872_2017_576_MOESM1_ESM.pptx]

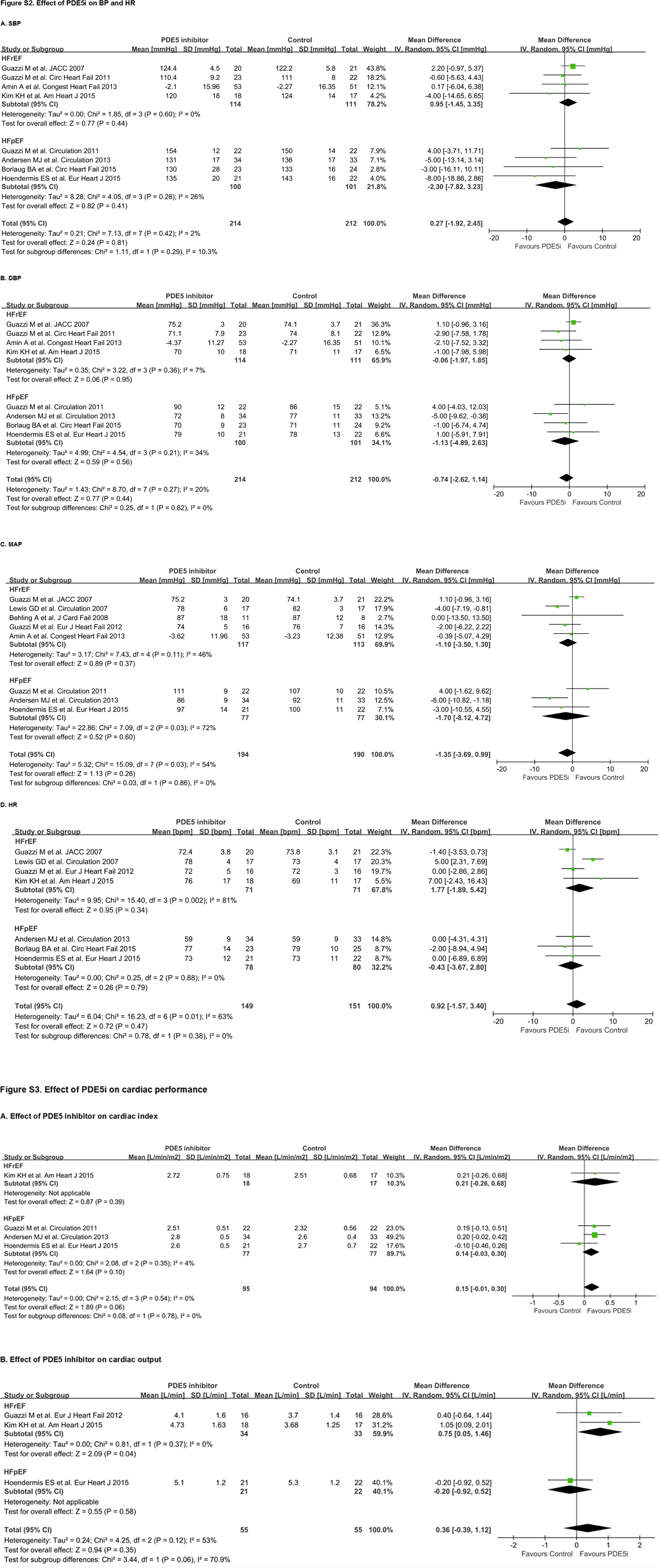

Supplement: Supplementary file 2 — Effect of PDE5i on BP and HR. Forest plot of the pooled weighted mean differences of (A) SBP (mmHg), (B) DBP (mmHg), (C) MAP (mmHg), and (D) HR (beat per minute). Abbreviations: SBP, systolic blood pressure; DBP, diastolic blood pressure; MAP, mean arterial pressure; HR, heart rate. Figure S3. Effect of PDE5i on cardiac performance. Forest plot of the pooled weighted mean differences of (A) cardiac index (L/min/m2), and (B) cardiac output (L/min). (TIF 5850 kb) [file 12872_2017_576_MOESM2_ESM.tif]

## Slide 1
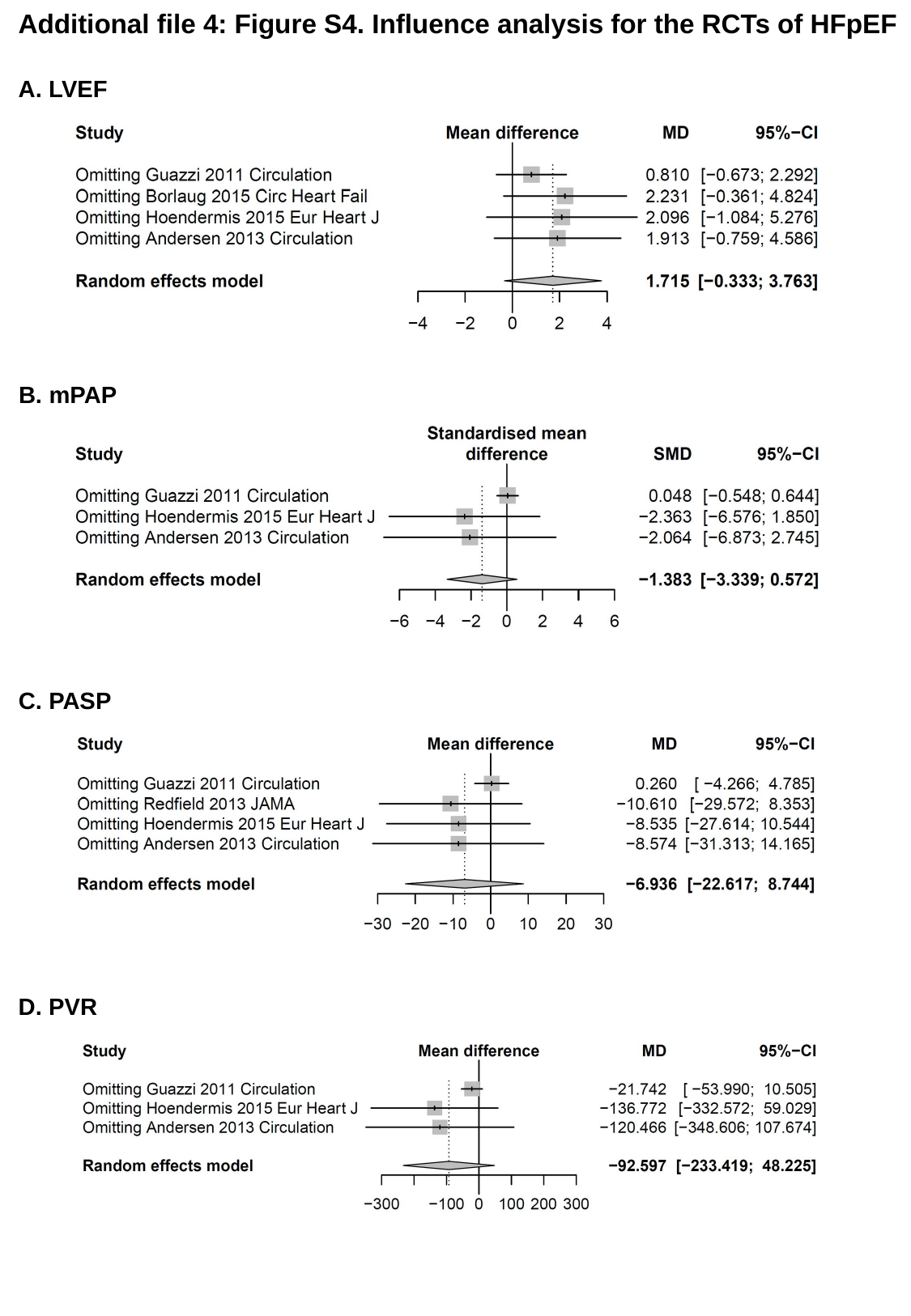

Additional file 4: Figure S4. Influence analysis for the RCTs of HFpEF
A. LVEF
B. mPAP
C. PASP
D. PVR

Supplement: Supplementary file 3 — Influence analysis for the RCTs of HFpEF. Sensitivity analysis was performed to assess the potential influence of each RCT to the effect size of the RCTs of HFpEF. Pooled effects of PDE5i when each RCT was omitted were shown for (A) LVEF (%), (B) mPAP (mmHg), (C) PASP (mmHg), and (D) PVR (dyn·sec/cm5). The omission of the study by Guazzi M et al. [12] significantly changed the pooled effect size of PDE5i, suggesting that there was a substantial influence from the study by Guazzi M et al. on the overall outcome measures. Abbreviations: RCT, randomized controlled trial; HFpEF, heart failure with preserved ejection fraction; PDE5i, phosphodiesterase type 5 inhibitor; LVEF, left ventricular ejection fraction; mPAP, mean pulmonary artery pressure; PASP, pulmonary artery systolic pressure; PVR, pulmonary vascular resistance. (PPTX 588 kb) [file 12872_2017_576_MOESM3_ESM.pptx]
